# Challenges for Management of Dilated Cardiomyopathy during COVID-19 Pandemic—A Telemedicine Application

**DOI:** 10.3390/jcm11247411

**Published:** 2022-12-14

**Authors:** Luminita Iliuta, Andreea Gabriella Andronesi, Eugenia Panaitescu, Madalina Elena Rac-Albu, Alexandru Scafa-Udriște, Horațiu Moldovan

**Affiliations:** 1 Medical Informatics and Biostatistics Department, University of Medicine and Pharmacy “Carol Davila”, 050474 Bucharest, Romania; 2 Cardioclass Clinic for Cardiovascular Disease, 031125 Bucharest, Romania; 3 Nephrology Department, University of Medicine and Pharmacy “Carol Davila”, 050474 Bucharest, Romania; 4 Nephrology Department, Fundeni Clinical Institute, 022328 Bucharest, Romania; 5 Department of Cardio-Thoracic Pathology, University of Medicine and Pharmacy “Carol Davila”, 050474 Bucharest, Romania; 6 Department of Cardiology, Clinical Emergency Hospital, 014461, Bucharest, Romania; 7 Department of Cardiovascular Surgery, Clinical Emergency Hospital, 014461 Bucharest, Romania; 8 Academy of Romanian Scientists (AOSR), 3 Ilfov Street, 050044 Bucharest, Romania

**Keywords:** remote monitoring, heart failure, COVID-19, telemedicine, dilated cardiomyopathy, diastolic dysfunction, restrictive pattern

## Abstract

Background and Objectives: The 2019 coronavirus pandemic (COVID-19) represented a significant challenge for the medical community. The first aim of this study was to examine the COVID-19 impact on the follow-up of patients with dilated cardiomyopathy (DCM) and to establish the advantages of multiparametric home monitoring. Also, we tried to establish the main prognostic predictors at 2-years follow-up and the value of LV diastolic filling pattern (LVDFP) in increasing mortality and morbidity. Materials and Methods: We conducted a prospective study of 142 patients with DCM assessed by in-patient visit in the pre-pandemic period and hybrid (face-to-face, online consultation and telemedicine home monitoring with a dedicated application) during the pandemic period. The statistical analysis compared the strategy used in the pre-pandemic with management during the pandemic, in terms of clinical assessment, hospitalizations/emergency room visits due to HF exacerbation and total mortality. Results: We did not observe significant changes in blood pressure (BP), heart rate (FC), weight and symptoms or an increased rate of adverse drug events between the two periods. We successfully titrated HF medications with close monitoring of HF decompensations, which were similar in number, but were mostly managed at home during the pandemic. There was also no statistically significant difference in emergency room visits due to severe decompensated HF. Mortality in the first and second year of follow-up was between 12.0 and 13%, similar in the pre-pandemic and pandemic periods, but significantly higher in patients with restrictive LVDFP. Clinical improvement or stability after 2 years was more frequent in patients with nonrestrictive LVDFP. The main prognostic predictors at 1 and 2-years follow-up were: the restrictive LVDFP, significantly dilated LV, comorbidities (DM, COPD), older age, associated severe mitral regurgitation and pulmonary hypertension. Conclusions: The pandemic restrictions determined a marked decrease of the healthcare use, but no significant change in the clinical status of DCM patients under multiparametric home monitoring. At 2-years follow-up, the presence of the restrictive LVDFP was associated with an increased risk of death and with a worse clinical status in DCM patients.

## 1. Introduction

Dilated cardiomyopathy (DCM) represents a significant cause of morbidity and mortality between patients with heart failure (HF) and aging population. Its evolution is often ondulatory and difficult to predict on short and long-term, especially in patients with multiple comorbidities [1,2]. Despite modern therapy and progresses on surgical treatment, the overall prognostic of this disease remains poor, with a 50% mortality at 5 years [3,4], comparable with that for some of the most frequent neoplasia. Cardiologist is in the position to choose between using different parameters in order to evaluate the severity and the prognosis of the disease and to improve therapeutic management [5].

On the other hand, the global pandemic Coronavirus disease 2019 (COVID-19) impacted significantly the patients’ access to telehealth-care system. All specialties were affected by the changes in clinical prioritization, but especially cardiac patients who often need close medical monitoring. In order to reduce viral transmission, many countries introduced lockdown, but these restrictions affected vulnerable patients, like those with HF. Due to advanced age and comorbidities, especially diabetes mellitus (DM), patients with HF have a higher risk of serious infections, including COVID-19, but we have to take into account that, during pandemic, their evaluation also was impacted by reduced social contacts, decreased physical activity and reduced access to healthcare.

In Romania, a state of national emergency was declared from 16 March to 14 May 2020, which implied functioning of only essential services and the advice of staying at home for the general population. The lockdown had a profound impact on the workflow of the hospitals. The entire healthcare system had to reorganize to withstand the pandemic and to find a solution for the care of high-risk patients, such as HF patients. Remote follow-up visits with the use in most cases of phone-calls for stable HF patients were recommended, and the direct patient-doctor contact was reserved for the emergencies.

In recent years, HF management has made important progress, focusing on treatment modalities based not only on traditional drugs, but on various devices to meet the requirements of this complex syndrome. Virtual care models of telemedicine are used to follow-up patients with cardiac implantable electronic devices (CIEDs) and home sensors (wireless pulmonary artery haemodynamic monitoring system) and they have already proved to be efficient in monitoring HF patients in different trials [6,7,8,9,10]. Also, there is increasing interest in expanding virtual CIED care models, including pacemakers, implantable cardioverter defibrillators, and cardiac resynchronization therapy to reduce mortality and hospitalizations in patients with HF. Unfortunately, not all countries benefit from the economic resources necessary for the large-scale application of these devices. In our country, there is a small percentage of patients with HF who benefit from remote monitoring devices, so that, in the context of the pandemic, access to cardiovascular care had to be based on classic remote monitoring. In this context, telemedicine is useful in reducing space and time barriers, thus increasing patients’ compliance. However, little is known about the importance of use of the telemedicine in pandemic or lockdown situations.

For this reasons, we aimed to characterize the impact of remote monitoring using a dedicated telemedicine application during the COVID-19 pandemic in our country in patients with DCM and without using interogation of implanted devices. We immediately adapted our clinic dedicated application in order to keep the follow-up of the stable patients with the minimum possible physical contact and we reserved the face-to-face evaluation and interventions for unstable patients. That is why, the first objective of our study was to examine the impact of the COVID-19 pandemic on the healthcare system use and on the clinical status and evolution of patients with DCM under multiparametric home monitoring in a cardiovascular clinic from Bucharest.

On the other hand, a lot of studies revealed that the presence of a restrictive LV diastolic filling pattern (LVDFP) involves a more unfavorable prognosis in most of cardiac diseases (coronary, valvular or congenital) [11,12,13]. Also, diastolic dysfunction seems to be one of the earliest detectable abnormalities in a lot of the heart disorders. In DCM, the long and medium-term prognosis is influenced by many parameters, amongst which LV diastolic performance is one of the most important [14]. The second purpose of this study was to establish the medium-term prognostic predictors and the implication of the LVDFP on the evolution of patients with DCM at 2-years follow-up.

## 2. Materials and Methods

### 2.1. Study Population, Setting, and Data Collection

We carried out a prospective study in 142 patients with DCM evaluated between 1 March 2019 and 1 March 2022. All patients were recorded in the dedicated application of the Cardioclass clinic for cardiovascular disease at least 1 month before the beginning of the national lockdown. Patients were eligible for enrolment if they were diagnosed with DCM and had been evaluated in our clinic and registered in the dedicated application within the previous 12 months. All patients included in this study signed the informed consent form (approved by the institutional ethics committee) in which they authorized the prospective collection of data for research purposes.

In the pre-pandemic period (from 1 March 2019 to 1 March 2020), patients’ standard follow-up consisted of in-person appointments (minimum of one appointment per trimester) with a cardiologist physician consultation, ecocardiographic examination and/or ambulatory electrocardiogram or blood pressure monitoring. All patients registered in our clinic had also access to a specific phone number (allocated to nursing staff) to contact the team in case of any warning sign or symptom using a self-monitoring register for vital signs, symptoms and weight. For this home-monitoring we provided before pandemic period for all our HF patients learning instruments, but the data obtained were not included in our dedicated application. We had also a dedicated email for the patients with HF in order to facilitate communication. In some cases, we scheduled follow-up phone calls to better evaluate the patients’ symptoms and eventually to adjust treatment, mainly the diuretics doses. Whenever it became necessary, patients with congestion signs and poor response to oral diuretics had been admitted to our clinic in order to administer intravenous diuretics, using a pre-specified protocol.

During the restricted-pandemic period (from 1 March 2020 to 1 March 2021), the face-to-face appointments were reduced drastically (with no visits in the lockdown till 15 May 2020), and there were limited to urgent situations and to patients in NYHA classes III/ambulatory IV (who were however fully evaluated face-to-face, including by echocardiography at least once a year). All appointments scheduled before pandemic period were changed to remote consultations (on-line or phone appointments, including drug prescriptions using email or short message service, with ambulatory adjustments of drugs) and with careful identification of the patients who would need an in-person care. In order to check for drug compliance, we monitored the need for drug prescription renewal. Also, most of the blood tests were performed locally, allowing home-based phlebotomies. More than half of the analysis were obtained directly from the laboratory that processed them. For the rest, the results were sent by email or by phone via WhatsApp to our nurses and entered in the dedicated application. In urgent cases, ambulatory monitoring of electrocardiography (ECG) and blood pressure and transthoracic echocardiograms (with a portable Vivid machine) were performed, but all the stress tests were cancelled.

During pandemic, all patients were home monitored with a multiparametric application (linked with our dedicated software platform) that included daily heart rate (HR), blood pressure (BP), body weight and symptom status. The receiving application could additionally incorporate blood test results and electrocardiograms (via Istel HR-2000 remote monitoring system). This application allows for weekly transmissions of all the monitored parameters and sometimes blood test results and electrocardiograms to the remote monitoring server. Collected data were evaluated and filtered by a specialized team of nurses and physicians in the application and relevant medication changes were communicated to the patients. Also, the monitored parameters were subject to alerts based on pre-specified cut-off absolute values or variations over time.

The main alerts for HF decompensation diagnosis were:

- weight gain more than 4 kg in comparison to the patient’s reference weight or weight gain more than 2 kg in five consecutive days;
- mean HR more than 100 b/min in three consecutive days
- increase of blood urea or NT-proBNP more than 30% from the last known value
- worsening of HF symptoms based on our dedicated questionnaire.

For simplification of monitoring process and the alerts system, the responses to questions related to HF symptoms were grouped and classified as Good, Attention and Alarm as follows:

Good—No shortness of breath at rest

- Weight gain in one day less than 1–1.5 kg
- No swelling of feet, ankles, legs or belly
- No chest pain
- Non restricted daily activities

Attention—Feel more tired or have worsening shortness of breath at activities or you need more pillows to sleep or you can only sleep while sitting

- Weight gain in one day more than 1–1.5 kg or more than 2.5 kg in one week
- Swelling of feet, ankles, legs or belly
- Dry cough
- You feel more depressed than usual

Alarm—Shortness of breath at rest

- You wake up at night because you cannot breathe
- Weight gain of more than 3.5 kg in one week
- Important swelling of feet, ankles, legs or belly
- You have pain, pressure or tightness in your chest
- You feel confused or dizzy and can’t think clearly

Also, through the dedicated application and during the “face-to-face visits”, we evaluated two aspects of the global quality of life using a self-reported questionnaire: the physical component (PCS) and the mental component (MCS). The allocated points started from 0 (lowest) up to 10 (highest quality of life). Patients evaluated their change in mental and physical quality of life by answering at the question: “How would you rate your quality of life now?”. They had to choose between “Better than previous visit,” “The same as previous visit,” and “Worse than previous visit”.

Visits at the emergency units due to the decompensations of the HF were defined as ambulatory day-admissions, with the administration of intravenous diuretics, while hospitalizations were considered in-hospital admissions for intravenous diuretics or admissions to the intensive care unit for administration of inotropic support.

In the relaxed-pandemic period (from 1 March 2021 to 1 March 2022), we provided a hybrid follow-up of the patients with in-person appointments (minimum of one appointment per year—which included a cardiologist consultation, ecocardiographic examination and/or ambulatory ECG or blood pressure monitoring) and telemedicine consultations (on-line or phone appointments and the use of the dedicated multiparametric application).

Variables of interest were compared between three periods: pre-pandemic (1 March 2019–1 March 2020), restricted-pandemic (1 March 2020, including during lockdown from 16 March to 14th of May 2020 and then till 1 March 2021) and during relaxed-pandemic period from 1 March 2021 to 1 March 2022.

Physiological variables and episodes of decompensations of the HF were evaluated at the enrollment and during the following three years. The telemedicine monitoring application collected data on symptoms, heart rate, weight, blood pressure, ECG, blood tests, HF decompensation episodes, changes in the prescriptions, other medical consultations, and details regarding potential hospitalizations. Moderately HF decompensations resulted in treatment adjustments, including diuretics augmentation.

### 2.2. Ultrasound Methods

The patients were evaluated by echocardiography at the enrollment (pre-pandemic) and during pandemic, at least one ecocardiographic evaluation every year. We used a Philips Affinity30 or a portable General Electric VIVID machine, with a 3.5 MHz probe for all examinations. All techniques and calculations respected the recommendations of the European and American Society of Echocardiography [14].

At each visit, the main parameters assessed were: dimensions of the heart cavities (left ventricle- LV- end-systolic and end-diastolic diameters and volume, left atrium- LA- diameters, including LA indexed volume) and LV systolic and diastolic performance (with all Tissue Doppler- TDI- parameters measurement) [15]. 

We used a modified Simpson’s method for left ventricular ejection fraction (LVEF) calculation and we record the transmitral flow placing the pulsed wave Doppler (PW) between the mitral leaflet in an apical 4-chamber view. Using PW evaluation, we measured the transmitral flow velocities (Peak Early Diastolic velocity—E wave and late Diastolic velocity—A wave) and the deceleration time (DT). For TDI measurements we placed the PW sample volume in the lateral mitral annulus in the same apical 4-chamber view. We recorded peak annular systolic velocity (Sa), early diastolic velocity (Ea) and late diastolic velocity (Aa).

We classified the LV diastolic performance using LVDFP as follows:

- normal LVDFP (E/A > 1, DT < 220 ms, IVRT = 60–100 ms, Ea/Aa > 1),
- impaired relaxation (E/A < 1, DT > 220 ms, IVRT > 100 ms, Ea/Aa < 1),
- pseudonormalization: (E/A = 1–2, DT = 150–200 ms, IVRT < 100 ms Ea/Aa < 1), and
- restrictive pattern (E/A > 2 or DT < 150 ms, IVRT < 60 ms, Ea/Aa < 1)

Before pandemic period, in order to diagnose the ischemic etiology of DCM, we performed coronary angiography or coronary angio-CT to all patients over 35 years of age, as well as for patients under 35 years old with angina pectoris. 69 patients had associated coronary artery disease (>50% reduction in luminal diameter of any coronary artery). BNP titration was done for all patients at least 3 times per year and for HF diagnosis we consider the age-independent cut-off of 300 pg/mL [16].

All patients were treated with the standard medication for HF including digitalis, diuretics, angiotensin converting enzyme inhibitors and spironolactone. At the moment of enrolment, all the patients were in sinus rhythm.

Depending on the LV systolic function the patients were divided in two groups:

(a) Group A—105 patients with a moderate LV systolic dysfunction (LVEF = 25–35%), and

(b) Group B—37 patients with a severe LV systolic dysfunction (LVEF ≤ 25%).

**Figure 1:**
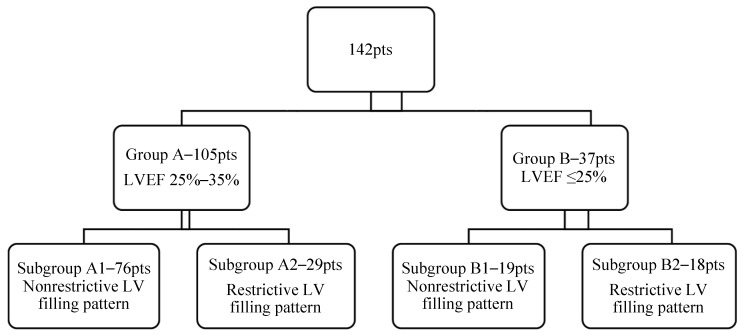
Study group structure depending on LV systolic and diastolic performance.

Depending on both systolic and diastolic function of the LV, each group was divided in two subgroups:

- Subgroup A1—76 patients with a nonrestrictive LVDFP
- Subgroup A2—29 patients with a restrictive LVDFP
- Subgroup B1—19 patients with a non-restrictive LV filling pattern, and
- Subgroup B2—18 patients with a restrictive LV filling pattern (Figure 1).

The demographic characteristics (mean age, gender), secondary mitral regurgitation degree and the mean pulmonary artery pressure were similar in the two study groups.

### 2.3. Statistical Analysis

We performed statistical analysis using Statistical Package for the Social Sciences, version 18.0 for the regression analysis and calculation of correlation coefficient and relative risk.

Categorical variables are expressed as absolute numbers (percentages). Normally distributed continuous variables are presented as mean ± standard deviation. Non-normal distributions are presented as median (interquartile range). The univariate comparison of baseline characteristics seen before pandemia and those seen during pandemia was made using the chi-squared test, Fisher exact test, Student’s *t*-test, or the Kruskal–Wallis test where appropriate. *p* value of <0.05 was considered statistically significant. Taking into account the exploratory nature of the study, for multiple comparisons adjustments were not made.

Categorical variables were tested with the χ2 test. To evaluate the homogeneity of variances Levene’s test was used. For testing the differences in mean values of continuous variables we used analysis of variance (ANOVA) with Games-Howell post hoc test for nonequal variances and Tukey post hoc test for equal variances. Wilcoxon test and Friedman test compared pre-pandemic and intra-pandemic variables. The association between pre-pandemic variables and the intra-pandemic change in hospitalizations and quality of life was based on Pearson correlation analysis, and logistic regression analysis established the association between pre-pandemic clinical and ecographical data and mortality or the magnitude of HF severity variation.

For the estimation of the medium-term prognosis the main endpoints used were: type of LVDFP, NYHA class for HF, quality of life and death.

## 3. Results

Demographic and Clinical Characteristics of the Patients

A total of 144 patients with DCM were eligible for the study; we excluded two patients because of missing data for the period of interest.

Information regarding the demographic, clinical, ecographic characteristics and HF therapies of the patients were obtained before the pandemic period, in the last appointment and is presented in Table 1. The majority of patients received target doses of HF medication in accordance with the current guidelines.

During the restrictive-pandemic period (2020–2021), there were only two patients diagnosed with COVID-19 who were infected in the hospital, with a mild form and favorable outcome (no cardiovascular or respiratory complications, with only mild symptoms). In the second year of follow-up, the percent of the vaccinated patients was more than 90%, and only 24 patients had a mild form of COVID-19.

In the pre-pandemic period, we registered a mean of 10 phone calls per month with a dedicated nurse for HF patients vs. a mean of 25 calls per month during the pandemic. Regarding clinical evaluation, in the pre-pandemic period all of the appointments were in clinic, with heart ultrasound evaluation during each visit. During the pandemic period, more than 80% of all the appointments were done remotely. During the second year of the pandemic period (relaxed-pandemic between March 2021 and March 2022), the number of the patients with in-clinic visits significantly increased compared to March 2020–2021 (restricted-pandemic)—78.17% vs. 19.71%, *p* < 0.05. 

We found that, despite the health care delivery barriers created by the COVID-19 pandemic, the use of telemedicine allowed us to not only continue monitoring patients with DCM, but also to expand the ambulatory treatment of HF decompensations and the cardiovascular counseling and treatment titration.

Even by remote appointments we were able to successfully titrate HF treatment, such as sacubitril/valsartan, with a careful monitoring of ambulatory blood tests results (serum creatinine, potassium and sodium levels), blood pressure, heart rate, weight and symptoms entered by the patients in the dedicated HF platform. There were no significant changes in weight, BP or HR between the study periods and no sustained ventricular arrhythmia occurred during any of the study periods.

The restricted and relaxed pandemic periods were not associated with significant changes in monitored parameters, including medium weight gain/month (1.2 ± 0.562 kg in pre-pandemic period versus 1.13 ± 0.657 kg during pandemic), systolic BP (121 ± 19 mmHg before pandemic versus 121 ± 18 during pandemic), HR (68 ± 10 b/min before pandemic versus 67 ± 10 b/min during pandemic, *p* = 0.05). Also, we did not observe an increased rate of adverse drug events during the online follow-up.

Regarding the self-reported patients’ quality of life score, there were no differences regarding the physical component (PCS) before and during pandemic. The mental component (MCS) of the self-reported quality of life was different in the three study periods. The percent of the patients with a favorable evolution quantified as a self-reported MCS more than five was significantly smaller during the restrictive pandemic period compared with pre-pandemic (80.28% vs. 24.65%) and increased during the relaxed-pandemic period (54.93%, *p* < 0.05).

The number of alerts before and during pandemic were similar. Overall, the remote monitoring center received a similar number of alerts during pandemic than before it, although the numbers of telephone calls were significantly higher during pandemic (3.6 ± 4.1 per patient before pandemic vs. 7.9 ± 3.2 during restricted-pandemic period and 4.6 ± 2.8 during relaxed-pandemic, *p* < 0.05). Blood tests were significantly reduced during the restricted pandemic and especially during the lockdown due to the important decrease in the laboratory visits. 

Before pandemic period, 31 patients reported HF decompensations and 29 of them required hospitalization. During restricted pandemic we observed 29 decompensated HF, but 21 pts were managed remotely by online consultation and 6 pts were admitted to our clinic in order to administer intravenous diuretics, using a pre-specified protocol. Most of the patients reported inability to access hospital. Also, more than 80% of the patients stated they would only attend hospital if there was no alternative. During relaxed pandemic there were 32 patients with decompensated HF from which 19 pts were managed online, 8 pts were treated to our clinic with intravenous diuretics and 5 pts needed hospitalisation.

Regarding emergency department visits due to significant HF decompensation, there was no statistically significant difference between the pre-pandemic and the pandemic period (*p* = 0.83).

The global mortality after the first year and second year of follow-up of this cohort (irrespective of the type of the LVDFP) was 11.97%, and 12.8% respectively, which was not significantly different compared to the mortality predicted by the Meta-Analysis Global Group in Chronic Heart Failure risk score at 1 year (mean value of 12.0% + 6.9%) [15]. Also, the number of deaths did not differ significantly in the pre-pandemic period compared to the pandemic.

Taking into account the type of the LVDFP, the mortality at one and 2-years follow-up was significantly higher in the restrictive LVDFP group (17.5% vs. 10.59% in the non-restrictive group for the first year of follow-up, *p* < 0.05, respectively 21.21% in restrictive group vs. 9.21% in the non-restrictive group for the second year of follow-up, *p* < 0.05), regardless of the LV systolic performance.

The presence of the restrictive LVDFP significantly increased the risk of death at 1 year and at 2-year follow up, irrespective of the presence of different parameters recognized to increase mortality in DCM patients. Regression analysis confirmed that the restrictive LVDFP was an independent predictor for increasing the risk of death or hospitalization for HF decompensations (*p* = 0.001), regardless of the LV dimensions or performance, the presence of a hemodynamically significant secondary MR or pulmonary hypertension. Furthermore, the prognosis of the patients with the restrictive pattern was worst, no matter of the other factors involved. 

**Figure 2:**
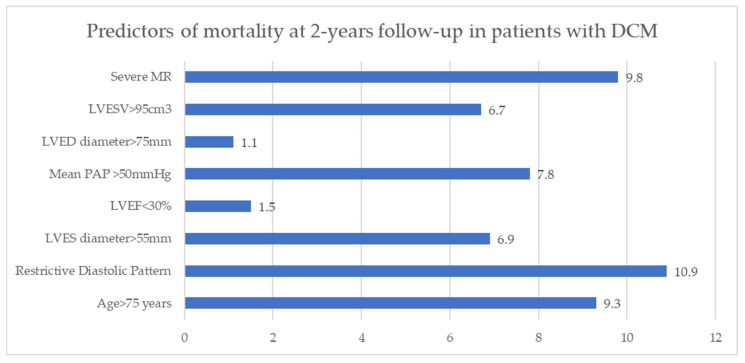
Predictors of mortality at 2-years follow-up in patients with DCM. MR—mitral regurgitation; LVESV—left ventricle end-systolic volume; LVEDD—left ventricle end-diastolic diameter; LVEDV—left ventricle end diastolic volume; LVESV—left ventricle end systolic volume PAP—pulmonary arterial pressure.

At 2-years follow-up in DCM patients the main parameters associated with unfavorable evolution revealed by multivariate logistic regression analysis were: patient’s age more than 75 years (RR = 9.3, *p* < 0.01), significantly dilated LV (end-systolic volume >95 cm3- RR = 6.7, *p* < 0.0001, end-systolic diameter > 55 mm—RR = 6.9, *p* < 0.05), restrictive LVDFP (RR = 10.9, *p* < 0.002), severe MR (RR = 9.8, *p* < 0.05) and severe pulmonary hypertension (RR = 7.8, *p* = 0.005) (Figure 2). 

MR- mitral regurgitation; LVESV- left ventricle end-systolic volume; LVEDD- left ventricle end-diastolic diameter; LVEDV- left ventricle end diastolic volume; LVESV- left ventricle end systolic volume PAP- pulmonary arterial pressure

Regarding the patients’ clinical course, the percentages of those with a favorable evolution quantified as NYHA class of HF less than 3 and self-reported quality of life score more than five at one- and two-years follow-up were higher in the nonrestrictive LVDFP group. At 1-year follow-up, the percentage of patients with a better or the same quality-of-life score was significantly higher in nonrestrictive LVDFP subgroup of patients compared with the restrictive one (58.13% vs. 13.04%, *p* < 0.005, likelihood ratio). Also, at 1-year follow-up, the percentage of patients in NYHA class less than 3 was four-fold in patients with nonrestrictive LVDFP (42.1% in nonrestrictive LVDFP group vs. 10.52% in restrictive LVDFP group, *p* < 0.05).

## 4. Discussion

The COVID-19 pandemic has posed a significant challenge to health systems in a multitude of ways. Patients with stable HF no longer had access to hospitals, these being reserved for seriously ill patients in emergency rooms and intensive care units. All clinics have had to adapt and pay attention to stable homebound patients and establish new remote strategies to continue providing quality care.

We found that despite the health-care delivery barriers due to COVID-19, telemedicine allowed us to continue seeing patients with DCM. This is the first study analyzing the impact of telemedicine on cardiovascular management during the COVID-19 pandemic in Romania. Our results support the ongoing use of telemedicine as a means to improve patient access to cardiovascular counseling and testing services.

Due to reduced healthcare contact and lifestyle changes, lockdowns could have a negative impact on HF patient. Our study suggest that our national lockdown had small impact on short-term in our HF patients who were adherent to remote monitoring. In countries with largely developed implanted electronic devices for HF patients (CIED), telemonitoring showed a significant and large reduction in hospitalisation, patients being managed with a wireless implantable haemodynamic monitoring system [17,18]. 

The TIM-HF2 (Telemedical Interventional Monitoring in Heart Failure) randomized trial proved that remote multiparametric management of patients with HF could reduce unplanned hospitalization rate and death [19]. On the other hand, BEAT-HF (The Better Effectiveness After Transition-Heart Failure) randomized clinical trial demonstrates that remote monitoring after discharge of hospitalized patients with HF using combined health coaching telephone calls and telemonitoring did not reduce 180-day readmissions [20]. 

We found a positive impact of our telemedicine model on the quality of life and the morbidity and mortality of DCM patients. We compared the data before and during the pandemic, in order to evaluate the impact of our telemedicine application. 

The COVID-19 pandemic determined significant anxiety amongst HF patients regarding cancellation of scheduled appointments, investigations, procedures, prescription, and monitoring services. Also, amplifying messages that those with chronic conditions should stay at home and avoid all physical contact may confuse and frighten HF patients, which may result in late presentation to the doctor in case of congestive symptoms. Even worse, patients with severe symptoms, whether due to COVID-19 or the underlying disease, may choose to stay at home with their family rather than risk isolation in the hospital.

During the pandemic, we intensified the number of remote visits and we were able to manage most of them without the need of hospitalization. There was no rise in emergency department visits and hospitalizations due to HF decompensation and no increase in mortality of all-cause, showing that this solution was safe and effective, and permitted also protection against SARS-CoV-2 infection. Due to the increase in telemedical management of HF patients, we were able to maintain a low rate of hospitalizations due to HF decompensation without an increase in mortality. In light of these results, we encourage the progressive use of telemedicine in patients with HF in a pandemic context, but also in situations where physical consultation is difficult for logistical reasons.

On the other hand, when we look at the study design, the first impression is that the HF patients received a lot more attention when they were assessed by telemedicine manners (weekly) compared to the face-to-face consultations (every three months). Although during the prepandemic period we monitored patients at least 1 visit per trimester, all patients registered in our clinic used a self-monitoring register for vital signs, symptoms and weight and had also access to a specific phone number and to a dedicated email (allocated to nursing staff) to contact the team in case of any warning sign or symptom. For this home-monitoring we provided before pandemic period for all our HF patients learning instruments, but the data obtained were not included before pandemic in our dedicated application. Also, even pre-pandemic, in some cases, we scheduled follow-up phone calls to better evaluate the patients’ symptoms and to adjust medication. Even so, since all these interventions were not clearly and timely quantified in the dedicated application, the question remains whether the positive results are not mainly because of the telemedicine pros, but because of the more detailed and frequent monitoring of the patients. We hope that, with the improved telemedicine application, in future studies we will be able to clearly clarify this question.

Anyway, with all the problems that the pandemic has involved, COVID-19 has highlighted unprecedented opportunities to expand telemedicine applications for monitoring patients at home. The development of interoperable telemedicine systems would also reduce geographical barriers, which are sometimes very important in the follow-up of patients with DCM. In addition, the time savings achieved through remote consultation offer flexibility to patients and may increase participation for those who are medically or socially vulnerable, or those who do not have immediate access to medical services.

The impact of telemedicine on doctors’ practices has been addressed in few previous studies. Positives noted included that telecommuting increased scheduling flexibility and physician availability to patients through online appointments. The negative aspects from the clinician’s point of view are related to the lack of face-to-face contact which, in certain situations is essential, and technological limitations. We also faced this in the study, where it was difficult to collaborate with rural or elderly patients.

Thus, to ensure that the delivery of telehealth care meets the needs of both patients and physicians, it is necessary to pay close attention to both technological barriers and human relationship needs. We believe that, by addressing these issues, the implementation of safe and effective virtual care on a large scale for patients with HF will be facilitated [21].

Our study reinforces what other studies have found. It is important to create telemedicine applications where patients are proactively enrolled and equipped with physiological monitors that can communicate vital information. Applications should include specific questions regarding symptomatology, quality of life, psychological health, physical activity and parameters specific to the chronic condition followed, all of which ensure the correct monitoring of patients and the prompt establishment of the indication for requesting a face-to-face evaluation.

On the other hand, data from the present study are in line with the results from other studies demonstrating that LV diastolic filling is an important predictor of severity and prognosis in DCM [22,23]. The restrictive LVDFP is frequently observed in DCM, especially in most severe forms of the disease, and is the best predictor for cardiac death [24,25,26,27]. Thus, in our study, the mortality rate at 2-year follow-up was significantly higher in patients with DCM with restrictive LVDFP compared with those with a nonrestrictive filling pattern. Evaluation by NYHA class and quality of life has shown that improvement at 2-years follow up was more frequent in patients with nonrestrictive LVDFP, same results being obtained in other studies [28,29,30,31,32].

The survival rate at 2-years was 94% in patients with a nonrestrictive filling pattern (defined as prolonged DT) compared to 52% for patients with restrictive LV diastolic filling [23]. The survival rate was 84%, 73% and 61% at 1, 2 and 4 years respectively in another study dealing with DCM patients, that is significantly lower compared to that of age- and gender-matched population. [26]. At the 2-years follow-up, in the group with standard treatment, we found a mortality rate slightly higher than those from the literature, probably because of the transplantation surgery which is not well developed in our country, and also because of the underuse of novel treatments, such as sacubitril.

The risk of death at 2-years follow-up was increased by the important enlargement of the LV, severe MR and severe pulmonary hypertension, as was showed in other previous studies [4,24,27,33,34,35]. 

We observed that, in spite of survival of patients with restrictive LVDFP, their quality of life was much worse compared to those with a non-restrictive pattern. Thus, severe systolic dysfunction of the LV has a less influence upon evolution compared to the restrictive LVDFP.

### Study Limitations 

There are some limitations of our study: it is a single-center observational study, with a small sample size, a medium follow-up period, and a low number of events.

Our study highlighted some of the limitations of telemedicine. 10% of patients failed to go to the testing laboratory, compared with in-person patient appointment when obtaining a patient blood sample was easier. Also, several older patients preferred in-person visits, despite their higher risk for COVID-19, probably due to a lower comfort level with telemedicine technology in older adults. We were unable to perform physical examinations via telemedicine. Also, because of the interoperability problems encountered in our country with patients’ medical records, the electronic registries were not systematized and there is no software that may generate an alert email every time one of the DCM patients is admitted to the hospital. 

We acknowledge that our results are based on experience gained during a health system crisis and do not represent the general experience in the practice of telemedicine. In our study we did not address the aspects of patient or doctor satisfaction with the dedicated telemedicine application developed in our clinic. Further studies could explore the prospects of expanding this application and the implications of the use of telemedicine over a longer period and not in pandemic crisis conditions both from the perspective of the doctor and the patient. In addition, at the time of designing the application, in full lockdown, we had no information available regarding the specific technical difficulties that might have arisen during remote visits.

Moreover, telemedicine may pose a significant ethical issue regarding protection of personal data, since it is possible that safety breaches may allow, at least in theory, external access to the database [36]. That is why, for the large-scale development of this dedicated telemedicine application, despite its effectiveness, it will have to answer all questions regarding ethical considerations (security and confidentiality of transmitted data, confidentiality and privacy of the patient-doctor relationship).

## 5. Conclusions

Despite a significant decrease in conventional measures of healthcare use, the clinical status of DCM patients under multiparametric monitoring was affected minimally by pandemic restrictions. This telemedicine strategy, combined with patient education, may reduce significantly and even cancel the health risks associated with strict lockdowns, as well as geographic barriers. The remote monitoring allowed early identification and home management of most of the HF decompensations during pandemic. Our findings suggest that a combination of home remote monitoring with in-patient visits represent a very good tool for preserving the care and safety of patients with DCM.

In patients with DCM, the presence of a restrictive LVDFP is associated with a more unfavorable prognosis, this type of filling increasing the risk of death. Also, restrictive filling involves a worsened clinical status of the patients (quantified as NYHA class and the quality of life).

At 2-years follow-up, the presence of a restrictive LVDFP, second- degree MR, dilated LV with LVESD >55 mm and LVESV > 95 mm^3^, and severe pulmonary hypertension can anticipate higher mortality rates in DCM patients.

## Figures and Tables

**Table 1:** Baseline clinical and demographic characteristics of the patients *n* = 142.

	Group A—105 pts LVEF = 25–35%	Group B—37 pts LVEF ≤ 25%	*p* Value
Mean (SD) age (years)	58 (12)	61 (11)	0.381 ^1^
Women	41 (39.05%)	15 (40.54%)	0.584 ^2^
Medical history, no. (%)			
Arterial hypertension	49 (46.66%)	18 (48.65%)	0.229 ^2^
Diabetes mellitus	55 (52.38%)	20 (54.05%)	0.135 ^2^
Paroxistic atrial fibrillation	42 (40%)	16(43.24%)	0.065 ^2^
Ischaemic aetiology of DCM, no. (%)	50 (47.62%)	19 (45.35%)	0.059 ^2^
Chronic kidney disease	35 (33.34%)	12 (32.43%)	0.338 ^2^
COPD	18 (17.14%)	7 (18.92%)	0.126 ^2^
Mean (SD) LVEF (%)	35 (5)	26 (4)	0.03 ^1^
Restrictive LVDFP	29 (27.62%)	18 (48.65%)	0.01 ^2^
Mean (SD) heart rate	75 (17)	74 (17)	0.64 ^1^
Systolic blood pressure (mm Hg)	125 ± 18	111 ± 12	0.052 ^1^
NYHA^a^ class I/II	40 (38.09%)	5 (13.51%)	0.001 ^3^
NYHA^a^ class III	52 (49.52%)	12 (32.43%)	
NYHA^a^ class IV	13 (12.638%)	20 (54.05%)	
Median NT-proBNP (IQR) ^b^ (pg/mL)	1192 (800–2693)	1929 (800–2693)	0.034 ^1^
Medications, no. (%)			
ACEi or ARB	67 (63.81%)	22 (59.46%)	0.114 ^3^
Sacubitril/valsartan	35 (33.34%)	11 (29.73%)	0.212 ^3^
Beta-blocker	98 (93.34%)	30 (81.08%)	0.071 ^3^
Mineralocorticoid receptor antagonist	99 (94.28%)	35 (94.59%)	0.511 ^3^
Ivabradine	10 (9.52%)	3 (8.11%)	0.442 ^3^
Digitalis	52 (49.52%)	29(78.38%)	0.001 ^3^
Diuretic	53 (50.48%)	37(100%)	0.001 ^3^
Implantable cardioverter-defibrillator	2 (1.90%)	3 (8.11%)	0.001 ^3^

LVEF—left ventricle ejection fraction; LV—left ventricle; NYHA—New York Heart Association; ACEi—angiotensin-converting enzyme inhibitor; ARB—angiotensin receptor blocker; COPD—chronic obstructive pulmonary disease. ^a^ New York Heart Association (NYHA) class reflects patients’ status in the last face-to-face prepandemic appointment. Plus–minus values are means ± standard deviation. ^1^ ANOVA; ^2^ Pearson chi-square; ^3^ Likelihood ratio. ^b^ NT-pro-BNP denotes N-terminal pro-B-type natriuretic peptide plasma levels expressed as pg/mL and IQR represents interquartile range.

## Data Availability

All data generated or analyzed during this study are included in this published article.

## Abbreviations

LVDFP—left ventricle diastolic filling pattern; DCM—dilated cardiomyopathy, LV—left ventricle, LVEF—left ventricle ejection fraction, DT—deceleration time, NYHA—New York Heart Association; ED—emergency department; DM—diabetes mellitus; COPD—chronic obstructive pulmonary disease; MR—mitral regurgitation; COVID 19—Coronavirus disease 2019; HR—heart rate; BP—blood pressure; HF—heart failure; ACEi—angiotensin-converting enzyme inhibitor; ARB—angiotensin receptor blocker; LVES—left ventricle end-systolic; LVEDD—left ventricle end-diastolic diameter; LVEDV—left ventricle end diastolic volume; PAP—pulmonary arterial pressure; EDt—E-wave deceleration time; IVRT—isovolumetric relaxation time; TDI—tissue doppler imaging.
